# Studies on the in vivo disposition of adriamycin in human tumours which exhibit different responses to the drug.

**DOI:** 10.1038/bjc.1986.141

**Published:** 1986-06

**Authors:** J. Cummings, C. S. McArdle


					
Br. J. Cancer (1986), 53, 835-838

Short Communication

Studies on the in vivo disposition of adriamycin in human
tumours which exhibit different responses to the drug

J. Cummings' & C.S. McArdle2

'Department of Medical Oncology, University of Glasgow, I Horselethill Road, Glasgow G12 9LX;
2Department of Surgery, Glasgow Royal Infirmary, Glasgow G4 OSF, UK.

In vivo disposition of Adriamycin (ADR) is
characterized by rapid uptake into most tissues and
long tissue transit time due to high affinity but
slowly reversible binding to nuclear DNA (Harris &
Gross, 1975; Terasaki et al., 1984). The role of drug
metabolism in this scheme has not been fully
elucidated (Chan et al., 1978). There is reason to
believe that uptake of ADR into solid tumours
behaves independently of the factors that govern its
distribution into tissues. For instance, ADR was
detected in only the outermost 4 to 6 layers of cells
of intra-abdominal ovarian tumours, indicating a
penetration problem not normally associated with
tissues (Ozols et al., 1979). Also, tumour cells
develop resistance to ADR believed to involve
active extrusion of the drug from cells (Dano,
1973). Studies on the uptake of ADR into solid
human tumours in vivo have been limited because
of the obvious problem of obtaining sufficient
numbers of specimens for meaningful evaluation.
Also, these studies have tended to compare drug
concentrations in tumours with unrelated tissues. In
this paper we have attempted to study several
aspects of the disposition of ADR in solid human
tumours in vivo: (a) dose/response, by sampling
from tumours with different known responses to
ADR; (b) tumour drug uptake, by comparing ADR
concentrations in tumours with adjacent normal
tissue and blood; (c) intracellular binding; and (d)
tumour metabolism.

Tumour and normal tissue specimens were
obtained from post-operative resections and open
biopsies from a total of 36 patients admitted to
Glasgow Royal Infirmary. Gastric carcinoma and
adjacent normal mucosa were from partial and full
gastrectomies; colorectal carcinoma and adjacent
normal colon mucosa were from anterior resections
and partial colectomies and breast carcinoma was
from breast lumpectomies. Breast cancer metastases
were from axilla node biopsies with evidence of
tumour nodules. Liver biopsy specimens from

Correspondence: J. Cummings.

Received 26 November 1985; and in revised form, 17
January 1986.

gastric and colorectal cancer patients and 5 ml of
venous blood from all patients were sampled
simultaneously with tumour resection. A low dose
of commercially  available  ADR   (25 mgm2,
Farmitalia, Milan, Italy) was administered i.v.
operatively  30 min  before  tumour  resection
(27 min +16 min s.d.). Resections and biopsies were
immediately taken to the pathology department
from histological sectioning and 1-2 g tumour
visibly clear of necrosis and normal tissue, 1-2 g
mucosa and 0.5 g liver were immediately frozen to
- 60?C with solid CO2 for determination of drug
and metabolite content. ADR and metabolite
concentrations of all samples were determined by
HPLC as previously described (Cummings, 1985).
Each tumour and tissue specimen was homogenised
and extracted using two different methods, both of
which employed Daunorubicin as internal standard
(Cummings et al., 1984; 1986). The first involved
mixing a 1 ml aliquot of homogenate directly with
5 ml of chloroform: propan-2-ol (2:1) for 30 min,
followed by centrifugation (I000g for 15min) to
separate three distinct phases. The upper aqueous
layer was discarded, the lower organic layer was
decanted over the middle tissue pellet, transferred
to a clean test tube and evaporated to dryness. The
residue was dissolved in a small volume of
methanol and injected on to the HPLC column.
Treatment of tissue homogenates directly with
organic solvents does not release bound ADR
(Ozols et al., 1979). Our direct extraction method
was used to determine the concentration of the
unbound ADR fraction in samples. The second
method involved pretreatment of a 1 ml aliquot of
homogenate with 0.2 ml of silver nitrate (33% w/v)
for 10 min at 40C before extraction with organic
solvent as described above. Silver nitrate releases
ADR bound to DNA by intercalation and
precipitates  proteins  (Schwartz,  1973).  We
combined pretreatment with silver nitrate and
organic solvent extraction to determine the
concentration of the free and reversibly bound
fraction of ADR and metabolites in samples. The
bound fraction, which was an indicator of
intracellular binding in samples, was then

- ) The Macmillan Press, 1986

836  J. CUMMINGS & C.S. McARDLE

calculated as a percentage by comparing recoveries
from both extraction techniques.

Results from all tumour and adjacent normal
tissue specimens were summarised and are
contained in Table I. There they are represented to
highlight the four areas under study: dose response
(published percentage response of patients to single
agent ADR vs. mean tumour ADR concentration);
uptake (mean intrapatient ratios of ADR
concentration in tumours over ADR concentration
in normal mucosa and serum); intracellular binding
(mean percentage of total concentration in tumour
and tissue bound to cellular components); and
metabolism. Results obtained from all liver biopsies
and blood samples are contained in Table II. The
mean concentration of ADR determined in
colorectal tumours (176+89s.d. ngg-1 tumour)
was significantly lower (P<0.001, Student's t test)
than  that   determined  in  breast  tumours
(819+482s.d. ngg-1 tumour) and gastric tumours
(659 + 231 s.d. ng g- 1 tumour). The levels of ADR in
colorectal tumours (73.1 + 38 s.d. percentage of
normal mucosa concentration) and to a lesser
extent in gastric tumours (47.7 + 18 s.d. percentage
of normal mucosa concentration) were close to
equilibrium with the levels of ADR in adjacent
normal mucosa. There was a strong correlation
(r = 0.95) between the mean concentration of ADR
in breast, gastric and colorectal tumours and the
percentage of patients that responded to single
agent ADR (not shown). When individual patient
ADR concentrations in tumours were compared
with individual ADR concentrations in serum, the
levels in the three tumours were many times higher,
indicating significant intracellular drug accumu-
lation in all cases. The mean concentration of ADR
determined in axillary node specimens was low
(55 + 48 s.d. ng g-1 tissue) and less than sera,
suggesting that at the time of sampling equilibrium
with blood borne ADR had not yet been achieved.
The determined fraction of the total ADR concen-
tration bound to intracellular components in all
tumour types was high (72-83%) and of the same
order of magnitude as the reported binding of
ADR to DNA in vitro and to tumour cells in
culture (Schwartz, 1983). The determined fraction
of the total ADR concentration bound to intra-
cellular components in normal tissue and liver was
significantly less than in tumours (P<0.01, Tables I
and II). There was no evidence of metabolites in
any one tumour and normal mucosa specimen,
although one metabolite has been reported to be
present in human tumour biopsies sampled at
different times from ours (Chan et al., 1978).
In liver, where the levels of ADR were
high  (5570+ 1500 s.d. ngg- ). two metabolites
were identified: adriamycinol 7-deoxyaglycone
(275 + 516 s.d.  ngg- 1)  and  adriamycin  7-

WI

0o

0
0

.A

0

"0
Lu

+1

.

C*.

00
.-

"0

C)
0.

C)
"0

*a

a
03

a
a

.C
a

Ut

CU

._

0.

0
C)

a   0

X6 . t  Qo

;s 0

0     -0

;6   a  Q  M

Xfl

z  SO X

0

Cn

Q

Lu

a  ..

CN      O

I.-O

.0

co      IRt

ON

en        1

C14

ch

= -l
CU  CU  C U -  o

.E 5. -5 a

0   C 0 0   '..
0 C) 0 0

U, p.   C,   w $,   C

co   C U3   U co   C)3

m      0   Q

I--
N-

I N1

Lu0

-4

0      ev

N-     00     Lu

(NI    -4     en
N-     00     00

(-I

ON
0-%

N

-"

1-1
00

.-
00

00
-
en

00

a-,
I  .-

CS
ct

a

C.

:Y

C.)

v

.

08
0
Rn

g

u)
to
CU
4

CA
Cd

00~
0

3

CU

CU

,0

g

CU
C)

0

,0

. e

-           00
en    CI   lt

(NI   00    I-

ON   NI      W)

L-    - 4

I--
00
CU"
00

DISPOSITION OF ADRIAMYCIN IN HUMAN TUMOURS  837

deoxyaglycone (65+18 s.d. nggt). There was a
marked inter-patient variation in liver concentration
of adriamycinol 7-deoxyaglycone, which is reflected
in the large value of s.d. (Table II). Metabolite
profiles in serum were different from liver.
Adriamycinol was detected in all patients and the 7-
deoxyaglycones in only 2-4 patients.

Studies of the in vivo disposition of ADR in
human tumours have been performed after repeated
sampling at a single time point (27min+ 16min
s.d.). For a single determination of a drug
concentration to yield information with which to
make comparisons, ideally plateau concentrations
should be measured. It is likely that plateau levels
are reached in the different tumours and tissues at
different times. Probably the best model available
to study what the kinetics of ADR may be like in
human tumours is the solid transplantable animal
tumour. Results from animal studies show that
peak levels are achieved in liver and intestine
almost immediately after i.v. administration, whilst
in tumours peak levels are achieved after 1-3 h
(Yesair et al., 1972; Yesair et al., 1980). At 30min,
the time chosen in this study, approximately 60-
100% of plateau levels are recorded in both normal
tissues and tumours.

In one of the few related studies, human tissue
and tumour specimens were obtained 1.5-4h after
i.v. administration of a dose of ADR similar to
that used in the present study (10-60mg m2, Chan
et al., 1978). In a single colon adenocarcinoma
sample, the concentration of ADR was 9.2pgg-1
4 h after drug administration. In a single breast
adenocarcinoma sample, the concentration of ADR
was 2.49 ig g- 1 2 h after drug administration. We
can offer no explanation for the large disparity
between these results and ours apart from the fact
that different analytical methodology was used
(TLC compared to HPLC) or at the time of
sampling a genuine difference existed. The main
finding of this study is that mean tumour drug
concentration correlated with percentage figures
published for response of each tumour type to
single agent ADR. The low levels of ADR in
colorectal tumours were not due to an inability to
accumulate the drug from the circulation.
Colorectal cancer, which is known to be refractory
to ADR (Moertel, 1975) may benefit from new
approaches in ADR cancer chemotherapy, such as
regional drug administration or carrier-mediated
drug targeting.

?00   S+

o N S :   ~ ~   ~

a)

C tQwS+

- 0

s -    ~

o

0i1-t

a)
'0

N     E+
o'

00'X I  B  +

o

Sk0

.0

E

We thank the Cancer Research Campaign for continued
financial support.

0o
0

CO

._

cd

CO4

a)

-0

+1

$M.

a)

Ct

0E

Cl

*C)

0=

0

*CT

41

P4

00 a)

a)

-a)

a)

IRt a)
>~1

838    J. CUMMINGS & C.S. McARDLE
References

CHAN, K.K., COHEN, J.L., GROSS, J.F. et al. (1978).

Prediction of Adriamycin disposition in cancer patients
using a physiologic, pharmacokinetic model. Cancer
Treat. Rep., 62, 1161.

CUMMINGS, J. (1985). Method for the determination of

4'-Deoxydoxorubicin, 4'-Deoxydoxorubicinol and their
7-deoxyaglycones in human serum by high-
performance liquid chromotgraphy. J. Chromatogr.
341, 401.

CUMMINGS, J., STUART, J.F.B., McARDLE, C.S. &

CALMAN, K.C. (1984). Identification of Adriamycin
and its metabolites in human and animal tissue and
blood. In Methodological Surveys in Biochemistry and
Analysis. 14, 245.

CUMMINGS, J., MERRY, S. & WILLMOTT, N. (1986).

Disposition kinetics of Adriamicin, Adriamycinol and
their 7-deoxyaglycones in AKR mice bearing a sub-
cutaneously growing Ridgway Osteogenic Sarcoma
(ROS). Eur. J. Cancer Clin. Oncol., 22, 451.

DANO, K. (1973). Active outward transport of

Daunomycin in Ehrlich ascites tumour cells. Biochim.
Biophys. Acta., 323, 466.

GOTTLIEB, J.J., RIVKIN, S., SPIGEL, S.C. et al. (1974).

Superiority of Adriamycin over oral nitrosoureas in
patients with advanced breast carcinoma. Cancer 33,
519.

HARRIS, P.A. & GROSS, J.F. (1975). Preliminary

pharmacokinetic model for Adriamycin (NSC-123127).
Cancer Chemother. Rep., 59, 819.

MOERTEL, C.G. (1975). Clinical management of advanced

gastrointestinal cancer. Cancer, 36, 675.

MOERTEL, C.G. (1976). Chemotherapy of gastrointestinal

cancer. Clin. Gastroenterol., 5, 777.

OZOLS, R.F., LOCKER, G.Y., DOROSHOW, J.H.,

GROTZINGER, K.R., MYERS, C.E. & YOUNG R.C.
(1979). Pharmacokinetics of Adriamycin and tissue
penetration in murine ovarian cancer. Cancer Res., 39,
3209.

SCHWARTZ, H.S. (1973). A fluorimetric assay for

Daunomycin and Adriamycin in animal tissues.
Biochem. Med., 7, 396.

SCHWARTZ, H.S. (1983). Mechanisms of selective

cytotoxicity of Adriamycin, Daunorubicin and related
anthracyclines. Topics Mol. Structure Biol., 3, 93.

TERASAKI, T., IGA, T., SUGIYAMA, Y. & HANANO, M.

(1984). Pharmacokinetic study on the mechanism of
tissue distribution of doxorubicin: interorgan and
interspecies variation of tissue-to-plasma partition
coefficients in rats, rabbits and guinea pigs. J. Pharm.
Sci., 73, 1359.

YESAIR, D.W., SCHWARTZBACH, E., SCHUCK, D.,

DENINE, E.P. & ASBELL, M.A. (1972). Comparative
pharmacokinetics of Daunomycin and Adriamycin in
several animal species. Cancer Res., 32, 1177.

YESAIR, D.W., THAYER, P.S., McNITT, S. & TEAGUE, K.

(1980). Comparative uptake, metabolism and retention
of anthracyclines by tumours growing in vitro and in
vivo. Eur. J. Cancer Clin. Oncol., 16, 901.

				


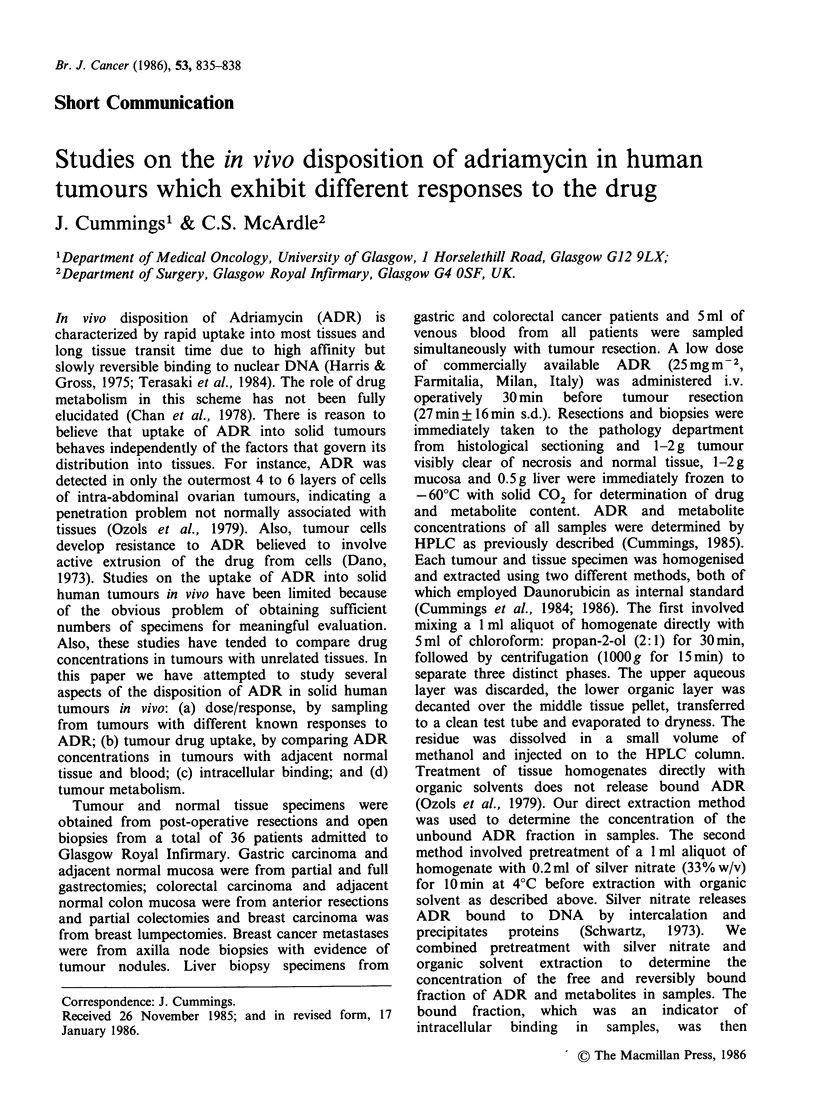

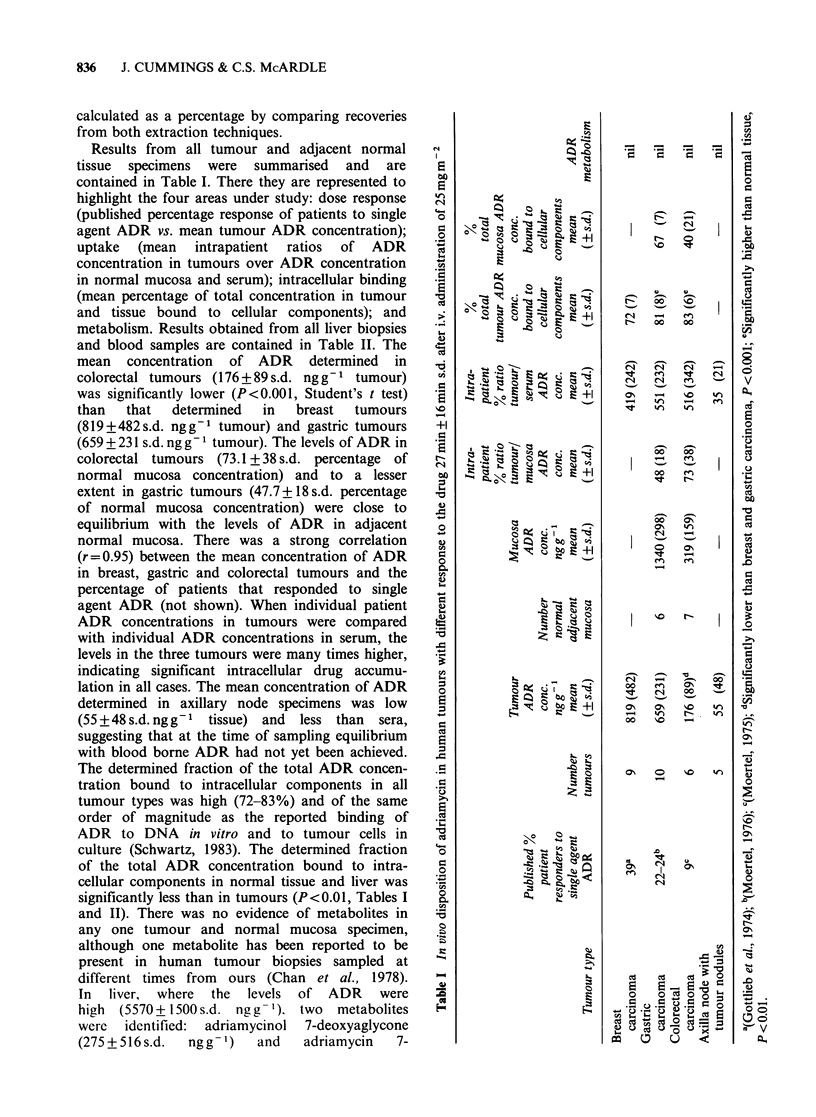

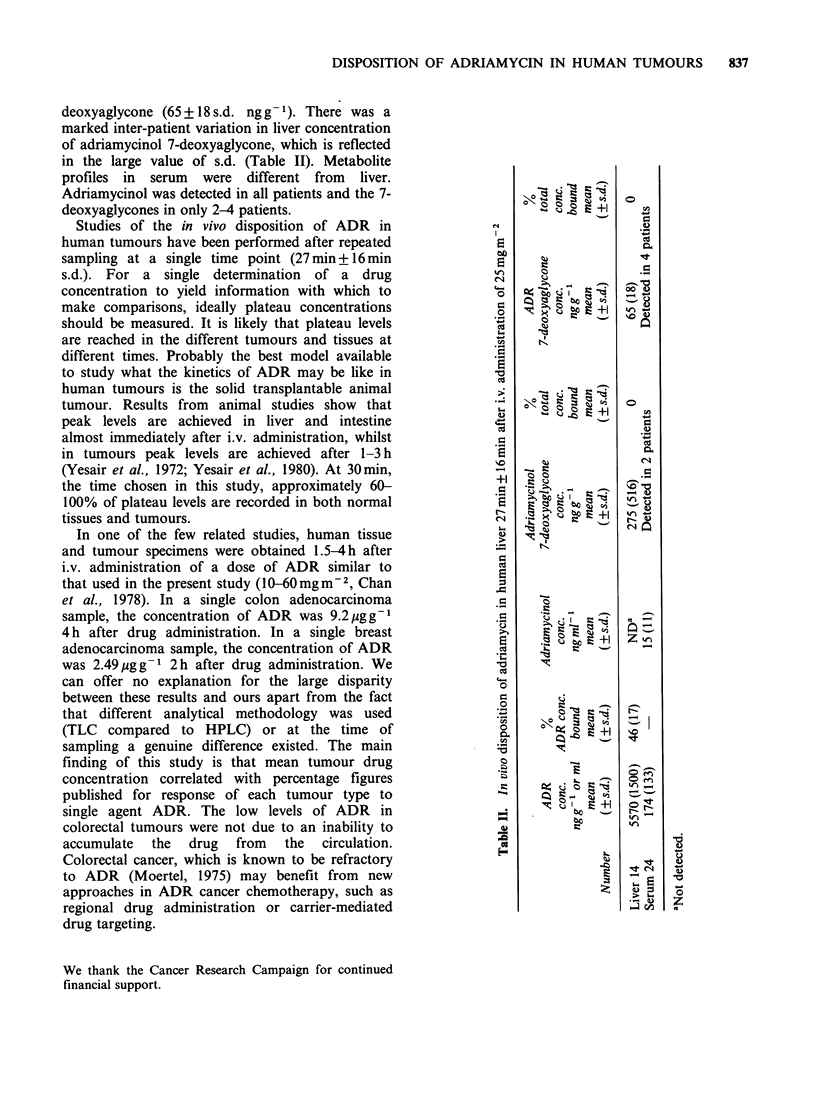

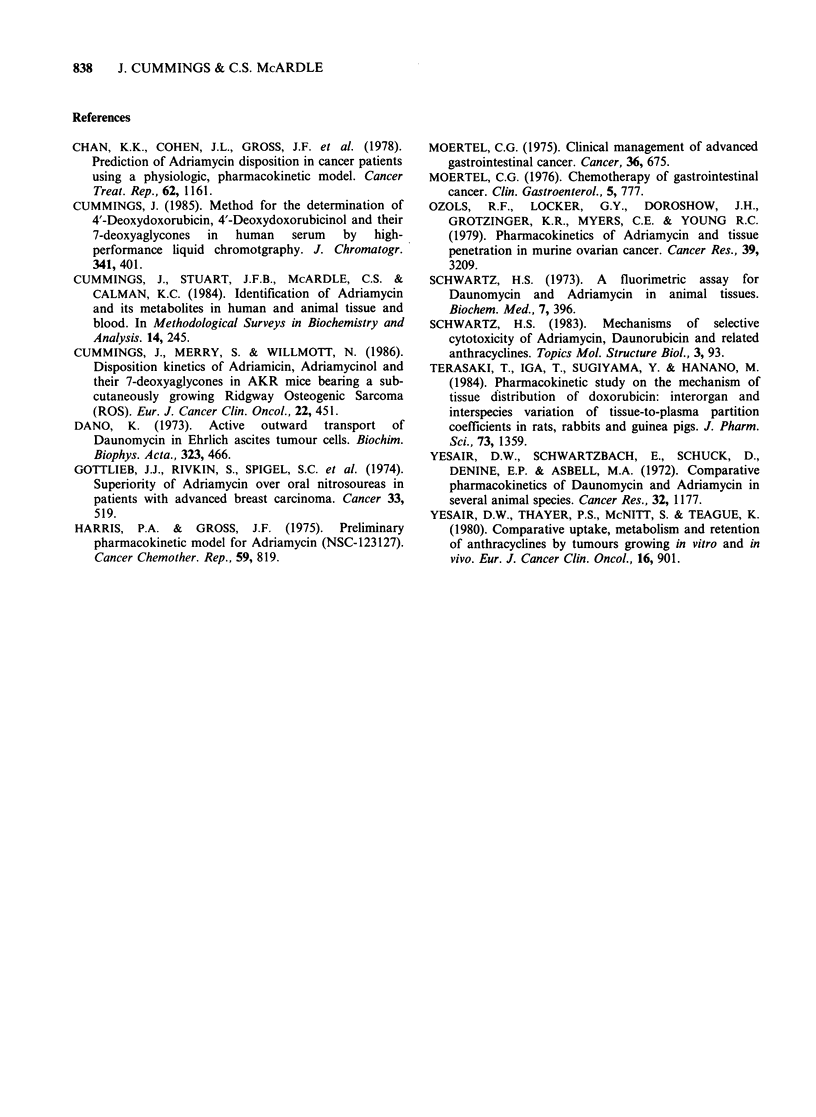

